# Evaluation of the Effect of Upper Complete Denture on Gustatory and Olfactory Senses

**DOI:** 10.5681/joddd.2009.032

**Published:** 2009-12-15

**Authors:** Tahereh Ghaffari, Fahimeh Hamedi Rad, Sepideh Mosadeg Kahnamoee

**Affiliations:** ^1^ Assistant Professor, Department of Prosthodontics, Faculty of Dentistry, Tabriz University of Medical Sciences, Tabriz, Iran; ^2^ Dentist, Private Practice, Tabriz, Iran

**Keywords:** Olfactory perception, taste perception, upper complete denture

## Abstract

**Background and aims:**

The majority of complete denture wearers are old. Clinical experience suggests that complete denture wearers have various disorders in their gustatory and olfactory senses due to disturbance of airways between the oral and nasal cavities caused by upper complete denture. The purpose of this study was to evaluate the effect of upper complete denture on gustatory and olfactory senses in denture wearers.

**Materials and methods:**

In this study, gustatory and olfactory senses in 30 patients (15 men and 15 women with a mean age of 52.93 ± 12.97 years) were evaluated three times: before complete denture insertion, three days after insertion and 1 month after that. Sucrose, citric acid, NaCl solution and distilled water (in two samples) were used to evaluate gustatory evalu-ation while mint- and cinnamon-flavored chewing gums were used for olfactory evaluation. Means and standard deviations were calculated and then compared between the different time intervals. P < 0.05 was considered significant.

**Results:**

The mean taste identification time was 5.23 ± 3.52 seconds before denture insertion, which decreased three days after insertion of denture and 1 month after use; however, these changes were not significant (P = 0.149). The mean time of flavor recognition increased after three days of insertion compared to the period before denture wear but decreased after 1 month; however, these changes were not significant (P = 0.792). In addition, the results revealed no significant differences in mean error in identification of taste and smell before and after denture insertion (P = 0.294).

**Conclusion:**

Denture wearing does not influence gustatory and olfactory senses.

## Introduction

Smell and taste disorders are common in the general population. Although these disorders may be signs of some underlying diseases and cause stress, depression and nutritional deficiency, they are overlooked by medical community.^1^ Patients try to overcome the problems by intake of greater amounts of salt and sugar, which would be dangerous for patients suffering from high blood pressure and diabetes.^2 , 3^Smell loss may result from nasal and sinus disease, upper respiratory tract infection or head trauma; common causes of taste disturbance include oral and perioral infections, oral appliances and Bell’s palsy.^1^ Taste receptors found within taste buds are located not only on tongue but also on the palate, pharynx, epiglottis, uvula and at the beginning of the esophagus.^4^ Repeated mechanical stimuli causes loss of taste receptors on palate in complete denture wearers.^5^

There are two pathways for olfactory sense: through the nostrils to the olfactory epithelium (orthonasal) and through the nasopharynx to their olfactory epithelium (retronasal). Odor is received through retronasal pathway at the liquid phase during eating and drinking. Most odors are received through the retronasal pathway, but the bulk of olfactory research has been designed to measure orthonasal olfactory sense and they have reported a disturbance in the elderly.^6^ There are few studies on retronasal olfactory pathway from foods in the mouth, although they have often compared the quality of olfactory sense in young and old individuals.^7 , 8^ The main complaint of complete denture wearers is disturbance in speech. Disorders pertaining to gustatory and olfactory senses have not been assessed yet.^8^

Clinical experience suggests that an upper removable denture might affect taste and smell by disturbing natural airflow between the oral and nasal cavities. Airflow is essential for the identification of retronasal flavor stimuli evoked during mastication and the upper removable denture prevents regular contact between the palatal receptor sites and taste samples.^4^

Patients seeking help because of a change in taste perception usually turn out to be suffering from an olfactory problem rather than an isolated gustatory problem.^9 , 10^ Since ‘taste’ and ‘flavor’ are synonyms in the current language, a decrease in flavor perception will lead to a complaint described as ‘taste loss’. This is mainly due to the influence of retronasal olfaction on flavor perception and underlines the importance of psychophysical taste and smell testing. Since taste and smell disorders may often occur simultaneously,,^12^ each chemosensory modality should be evaluated separately before drawing any conclusions.

Few studies in this area have, however, been carried out by orthodontic appliances.^4^ The present study was designed to evaluate the possible influence of complete dentures on the retronasal olfaction and the importance of psychophysical taste and smell testing.

## Materials and methods

This interventional study was performed in the Department of Prosthodontics at the Faculty of Dentistry in Tabriz University of Medical Sciences in 2009. According to a similar study,^4^ and statistical evaluation, 30 patients who needed upper complete dentures were included in this study. All the subjects were free of systemic diseases, had not had previous upper denture treatment, had not used any kind of drugs and at the time of examination did not suffer from any acute problems or diseases in their upper respiratory tract.

To assess taste recognition, each subject received a series of eight 5-mL samples representing tasteless, sweet, salty and sour substances in a random order. All intraoral stimuli were presented in disposable plastic cups at room temperature as follows:

Sucrose (Panizfam Co, Tehran, Iran) in 0.3 and 0.03 mol concentrations (labeled as stimulants A and B, respectively); citric acid (Mahram Co, Gazvin, Iran) in 0.24 and 0.024 mol concentrations (labeled as stimulants C and D, respectively); NaCl (Sepid Shayan Co, Shiraz, Iran) in 0.9 and 0.09 mol concentrations (labeled as stimulants E and F, respectively); and distilled water was presented in two samples (labeled as stimulants G and H). To assess flavor recognition as retronasal stimuli, two samples of chewing gum (ShirinAsal Food Ind Group, Tabriz, Iran) were chosen. These were of identical texture, color and hardness but differed only in flavor: mint and cinnamon.

All the tests were performed in a dental chair. The samples were presented to the subjects in an individual, randomized sequence. The patients were instructed to keep the solutions or chewing gums in their mouths until taste or flavor was identified. The elapsed time between the entrance of the samples into the mouth and identification by the patient was measured with a stopwatch and recorded by the examiner (accuracy up to 0.5 seconds). Between each of the samples the subjects rinsed their mouth with tap water. The mean duration of the whole testing session was approximately 6 minutes. In each session the participants were requested to do the following:

1. to write down the description of the taste or flavor in their own words (verbal labeling);
2. to mark the intensity on a visual analogous scale (VAS). The participants were asked to mark their answers on forms with 10-cm VAS. The VAS was used to record intensity estimates with endpoints marked by anchor statements of ‘strongest’ on the right and ‘weakest’ on the left.

Tests were performed in three sessions: once before denture insertion at least 1 week before taking final impressions (T0); three days after denture insertion (T1); and finally 1 month after that (T2). Only patients who replied correct answers in T0 session passed T1 and T2 (to ensure they had no disturbance in the gustatory and olfactory senses). If patients did not recognize the G and H stimulants as distilled water and or the distance between the two samples on VAS was more than 7 millimeters, they did not pass on.

Data was evaluated by comparative statistical methods (percentage, mean and standard deviation) using SPSS 13. Mean time of flavor recognition was analyzed by ANOVA. Accuracy of answers was analyzed by X^2^ and gustatory recognition by chi-square. The means and standard deviations in taste and flavor recognition times were calculated. The confidence level was established at P < 0.05.

## Results

In this study 30 patients who had not worn upper complete denture were chosen.

They consisted of 15 (50%) men with an average age of 52.64 ± 0.47 years and 15 women with a mean age of 53.21 ± 0.32 years, with no statistically significant difference.

**Figure 1:**
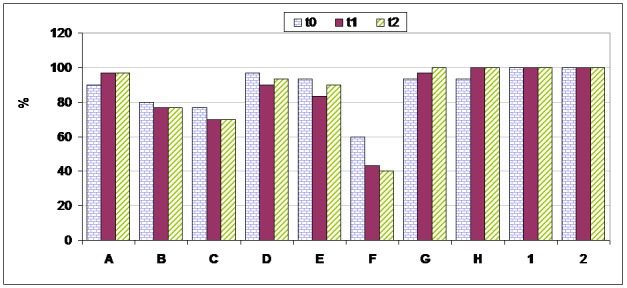
Mean percentage of correct verbal labeling for the various stimuli in the experimental group in the testing sessions.

*Correct verbal labeling of taste and flavor stimuli (Figure 1)*

The majority of stimuli were labeled correctly by the subjects. The most accurate identification was observed in the flavor stimuli (1and 2) and distilled water (G and H), while the most erroneous identification was found in the low-concentration NaCl solution (f). No significant differences were found between the 3 sessions regarding correct labeling.

**Figure 2:**
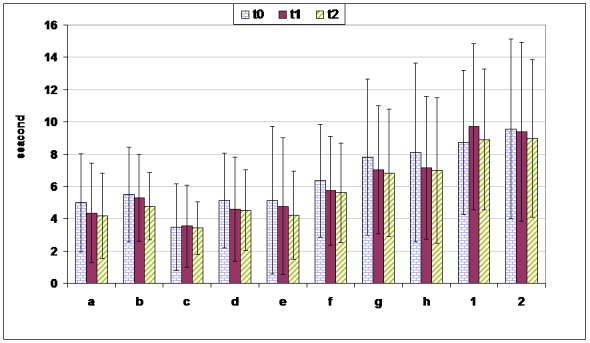
Mean identification time in seconds (± SD) for the taste and flavor stimuli in the experimental groups of the three testing sessions.

*Reaction time (Figure 2)*

The duration of reaction time between the stimulus presentation and the verbal report showed marked inter-individual variations. On average, taste stimuli were labeled within 3.5-8.1 seconds, while flavor identification required 8.9-9.5 seconds. The longest latencies for the various taste and flavor stimuli were measured at the first and second testing sessions, respectively. However, the mean reaction time for the different stimuli showed no significant differences between the 3 sessions (P = 0.294).

## Discussion

Based on prosthetic clinical reports it can be assumed that the presence of a removable appliance covering the palate could disturb normal oral functions, including smell and taste.^13 , 14^ Opinions differ regarding the location of taste buds. Schiffman^15^ indicated that no taste buds can be found in the area covered by the UCD (upper complete denture), while others claim that some gustatory ability can be found on the border between the soft and hard palate.^16^ The presence of UCD also prevents contact of the tongue with the palatal rugae. This contact is considered important by some researchers for dispersing the test sample and bringing it into a more appropriate contact with taste buds.^15^ Finally, the bulk of UCD may interfere with the normal mobility of the tongue and cheeks. This may prevent the release of flavors from the food samples and the free movement of the humid and warm air in the oral and nasal cavities, affecting retronasal olfaction.^17 , 18 , 19^

The number of taste buds decreases with age, resulting in an increase in gustatory threshold level.^2^Most denture wearers are old and several investigations reveal that gustatory ability in the elderly with complete denture is weaker than the youth,^3^ but it is not known how much of this weakness is due to complete denture.

Since repeated mechanical stimulation by upper complete denture results in the loss of taste nerve endings in the palate,^5^ patients with no previous complete denture treatment were chosen in the present study. They should have had no systemic disease because of the effect of these diseases on gustatory and olfactory abilities. Two samples of test substances were distilled water for testing participant's normal gustatory ability. Therefore, it is possible to claim that confounding factors were circumvented in this study.

In general, however, no significant differences were found between the 3 sessions, indicating that a UCD does not influence the patient's ability to detect and identify taste and flavors. Several aspects of these clear-cut results require further analysis.

The longer time needed for correct identification of the flavor stimuli might be attributed to the physical properties of the chewing gum as opposed to the liquid taste samples. In order to obtain a satisfactory intra-oral flavor stimulus, the chewing gum had to be manipulated for a certain period of time. This time period could not be decreased significantly with subsequent tests in the patients. Flavor identification time increased in T1 compared to T0, but decreased in T2. This can be explained by the more difficult oral handling of chewing gum when a patient uses new complete dentures for the first time. In Har-zion et al’s study there were decreases in T2 and T3 compared to T0.^4^

The reaction time for taste stimuli decreased consistently as the subjects learned the testing routine (Figure 2). In general, among six taste samples the mean minimum and maximum taste identification times were related to 0.24-mol citric acid in T0 and 0.09-mol NaCl in T0, respectively. In addition, the mean identification times in T0 and T1 were 5.23 ± 3.52 and 4.80 ± 3.40 seconds, respectively, which decreased to 4.54 ± 2.63 in T2; however, the differences were not statistically significant. Therefore, it can be claimed that denture wearing has no effect on gustatory abilities.

In our study as Har-zion et al’s study, the longest taste identification time was related to T0 and the least identification time was related to 0.24-mol citric acid. Maximum taste identification time in our and their study was related to 0.09-mol NaCl and 0.3-mol sucrose, respectively. In our study identification times in all the specimens decreased as concentration decreased; but in their study taste identification time for 0.3-mol sucrose was longer than that with 0.03-mol sucrose.^4^

In our study as Nilsson's study in 1979,^16^ the mean taste identification time in women was shorter than that in men because women’s gustatory ability is stronger than men.

How can these results be reconciled with the taste disturbances reported in patients using full dentures? The age of the patients may play an important role, as denture wearers in most cases are elderly and a well-known phenomenon in this age group is the decline in orthonasal and retronasal smell as well as in taste.

## Conclusion

The results indicate that a complete denture does not interfere with the taste and flavor sensations evoked by the stimuli used in this study.
